# Development of an Oral IgA Response against SARS-CoV-2 Following Immunization with Different COVID-19 Vaccines

**DOI:** 10.3390/v15122319

**Published:** 2023-11-25

**Authors:** Irene Soffritti, Maria D’Accolti, Francesca Bini, Eleonora Mazziga, Davide Proietto, Beatrice Dallan, Martina De Laurentis, Sara Ghisellini, Francesco Nicoli, Elisabetta Caselli

**Affiliations:** 1 Department of Chemical, Pharmaceutical and Agricultural Sciences, and Laboratorio per le Tecnologie delle Terapie Avanzate (LTTA), Section of Microbiology, University of Ferrara, 44121 Ferrara, Italy; irene.soffritti@unife.it (I.S.); maria.daccolti@unife.it (M.D.); francesca.bini@unife.it (F.B.); eleonora.mazziga@unife.it (E.M.); 2 Department of Chemical, Pharmaceutical and Agricultural Sciences, Laboratory of Biochemistry, Immunology and Microbiology (BIM), University of Ferrara, 44123 Ferrara, Italyfrancesco.nicoli@unife.it (F.N.); 3 Laboratory of Clinical Pathology, University Hospital St. Anna, 44121 Ferrara, Italy

**Keywords:** SARS-CoV-2, COVID-19, IgA, oral mucosal immunity, vaccine

## Abstract

The mucosal immune response is recognized to be important in the early control of infection sustained by viruses with mucosal tissues as the primary site of entry and replication, such as SARS-CoV-2. Mucosal IgA has been consistently reported in the mouth and eye of SARS-CoV-2 infected subjects, where it correlated inversely with COVID-19 symptom severity. Yet, there is still scarce information on the comparative ability of the diverse SARS-CoV-2 vaccines to induce local IgA responses at the virus entry site. Thus, the aim of this study was to assess the presence of anti-SARS-CoV-2 IgA in the saliva of 95 subjects vaccinated with a booster dose and different combinations of vaccines, including mRNA-1273 (Moderna), BNT162b2 (Pfizer-BioNTech), and Vaxzevria (AstraZeneca). The results showed the presence of a mucosal response in 93.7% of vaccinated subjects, with a mean IgA titer of 351.5 ± 31.77 U/mL, strongly correlating with the serum anti-SARS-CoV-2 IgG titer (*p* < 0.0001). No statistically significant differences emerged between the vaccine types, although the salivary IgA titer appeared slightly higher after receiving a booster dose of the mRNA-1273 vaccine (Moderna) following two doses of BNT162b2 (Pfizer-BioNTech), compared to the other vaccine combinations. These data confirm what was previously reported at the eye level and suggest that monitoring salivary IgA may be a useful tool for driving forward vaccine design and surveillance strategies, potentially leading to novel routes of vaccine administration and boosting.

## 1. Introduction

Coronavirus disease 19 (COVID-19), caused by severe acute respiratory syndrome coronavirus 2 (SARS-CoV-2) infection, has been recently declared as an ended international health emergency by the World Health Organization (WHO) [1]. However, despite this encouraging data, the infection continues to cause more than one thousand weekly reported deaths and an increasing number of cases of long-COVID syndrome are being documented [2,3]. The three-year emergency condition has driven research worldwide focused on understanding viral pathogenesis and transmission and the development of highly effective vaccines. Vaccination against severe acute respiratory syndrome coronavirus 2 (SARS-CoV-2) represented a crucial milestone in reducing the incidence, severity, and transmission of COVID-19. Indeed, the reduced risk to human health was mainly driven by the high level of immunization derived from infection and vaccination. To date (3 November 2023), 13.53 billion doses of COVID-19 vaccines have been administered globally, and more than 80% among priority groups (health workers and adults aged over 60 years) have completed the primary series of vaccination, although coverage varies in different world regions [2]. Several vaccination approaches have been developed to prevent SARS-CoV-2 infection, including those based on a non-replicating adenoviral vector, such as the ChAdOx1 Vaxzevria vaccine (AstraZeneca, Cambridge, UK), and mRNA-based vaccines, including Pfizer-BioNTech’s BNT162b2 (Mainz, Germany) and Moderna’s mRNA-1273 (Massachusetts, USA) vaccines, which have emerged as quick and efficient strategies since December 2020, when the US Food and Drug Administration (FDA) approved their emergency use [4,5]. 

Reported data showed that the administration of two vaccine doses was highly protective against COVID-19 and able to stimulate systemic antibody and cellular responses [6,7,8], showing a 95% efficacy in preventing the symptomatic disease [6]. However, the primary immunization showed a temporary effect over time, with a 50% loss of effectiveness and a parallel increased risk of acquiring SARS-CoV-2 infection after 6 months from the second dose [9,10]. The waning effectiveness of the primary vaccination, together with the emerging prevalence of variants of concern (VOC), including the B.1.617.2 (Delta), and B.1.1.529 (Omicron) variants, led to plans involving the administration of a third dose of the COVID-19 vaccine (booster) scheduled 5–6 months after the second dose [11]. A global analysis of booster effectiveness estimates a 93% efficacy in preventing COVID-19-related hospital admission, a 92% effectiveness in preventing severe COVID-19, and an 81% effectiveness in avoiding related deaths [12].

Since SARS-CoV-2 is mainly spread by airborne transmission, the oral mucosa, respiratory tract, and ocular surfaces represent the primary routes of viral entry, mediated by the interaction of the viral spike protein S1 with its angiotensin-converting enzyme 2 (ACE2) receptor [13]. Consistent with this, an increasing body of evidence supports a pivotal role of the mucosal immune response in the early control of SARS-CoV-2 infection [13,14]. 

Secretory type A immunoglobulins (IgAs) are the predominant antibody isotype found in saliva and other mucosal sites and serve as the primary defense mechanism against pathogens invading the mucosal surfaces of the respiratory and gastrointestinal tracts [15]. Several data report that SARS-CoV-2 infection is able to elicit mucosal immune responses, inducing the production of sIgA in mucosal secretions including bronchoalveolar lavage fluids, saliva, nasal fluid, and tears [16,17,18]. Anti-SARS-CoV-2 sIgAs were proven to effectively neutralize viral entry at the mucosal surfaces by binding the virus’s S1 protein and competitively blocking its interaction with the ACE2 receptor [13]. We were among the first to report the presence of anti-SARS-CoV-2 sIgA in the saliva of COVID-19 patients, showing that sIgA levels were inversely correlated with patients’ symptom severity and oral inflammation, thus supporting the potential protective role of the sIgA response against COVID-19 progression [19]. In addition to the oral site, we previously detected sIgAs at the eye level in the conjunctival fluid of about 40% of COVID-19 patients where they were maintained for up to 48 days post COVID-19 onset and whose titer was similarly associated with a mild course of the disease compared to patients with low or no detectable sIgA response in their conjunctival fluid [16]. More recently, we and others also reported the detection of a specific sIgA response at the ocular level following intramuscular SARS-CoV-2 vaccination [20,21].

While much research has focused on systemic antibody and cellular responses following COVID-19 vaccination, the investigation of mucosal immune responses, particularly salivary IgA levels, remains limited. Instead, recent data show that understanding the dynamics of salivary IgA levels following the booster dose of the anti-SARS-CoV-2 vaccine may provide valuable insights into the mucosal immune response and its potential contribution to enhanced protection against the onset of COVID-19 [22]. Consistent with this, the ability of anti-SARS-CoV-2 vaccines to induce effective mucosal immunity has recently become a topic of increasing interest [23,24]. 

Recent studies reported that SARS-CoV-2 vaccines can variably boost mucosal IgA responses which correlate with protection against subsequent COVID-19 infection [25]. Specific salivary IgG and IgA responses have been observed after the first cycle of BNT162b2 mRNA vaccination, although these antibodies did not display relevant neutralization activity, particularly in subjects not previously exposed to natural SARS-CoV-2 infection [26]. Salivary IgA was reported to be increased after eight months from the second dose, suggesting a delayed activation of mucosal immunity [24]. The BNT162b2 booster vaccine was reported to induce a strong systemic immune response compared with the primary vaccination cycle, with higher titers of both IgG and IgA in serum and saliva. Moreover, salivary neutralizing activity was highly effective against the Wuhan wild type strain, but less effective against the emerging VOC [24]. In another recent study, the effect of booster dose was investigated both in tears and saliva, pointing out that sIgA levels were significantly lower following booster vaccination compared to after a naturally occurring SARS-CoV-2 infection [8].

Since there are little data available to date on the mucosal immune response following booster vaccination, essentially referring to BNT162b2 (Pfizer-BioNTech) booster dose administration, the aim of this study was to characterize salivary IgA mucosal immunity in response to different combinations of COVID-19 vaccine formulations. 

## 2. Materials and Methods

### 2.1. Study Design and Population

An observational cohort study was performed at the University Hospital of Ferrara (Italy), after approval by the Ethical Committee of the Istituto Nazionale per le Malattie Infettive Lazzaro Spallanzani (protocol No. 65, approved on 28 April 2022), in order to evaluate the mucosal anti-SARS-CoV-2 IgA response elicited by booster vaccination. A total of 105 subjects were recruited on a voluntary basis 4–9 months after receiving the booster dose, during the period between February 2022 and September 2022. The inclusion criteria were: age over 18 years old, having received the third (booster) dose of the anti-SARS-CoV-2 vaccine, feasibility, and agreeing to provide saliva samples. The exclusion criteria included pregnancy, being under pharmacological treatment with immunosuppressive drugs, and ongoing autoimmune or neoplastic diseases. 

All recruited subjects received a primary vaccination cycle consisting of two doses of recombinant adenovirus-based Vaxzevria vaccine (AstraZeneca, A) or mRNA-based monovalent Comirnaty vaccine (Pfizer-BioNTech, P). In addition, all enrolled subjects received an mRNA-based vaccine booster dose, consisting of the Comirnaty (P) or Spikevax-1273 (Moderna, M) vaccine. Overall, the study population included four groups based on the following combinations of vaccines received: A-A-M, A-A-P, P-P-M, and P-P-P. 

All recruited subjects completed a self-report medical history questionnaire and signed an informed consent form. Demographic features were collected at recruitment, including age, gender, body mass index (BMI), and occurrence of SARS-CoV-2 infection. Also, laboratory data about circulating B and CD4^+^ lymphocyte number and anti-SARS-CoV-2 serum IgG titer were recorded at enrollment. The number of B and CD4^+^ T cells was determined in whole fresh blood stained with anti-CD4 PE-Cy7 (clone RM4-5, Thermo Fisher Scientific, Waltham, MA, USA) and anti-CD19 VioGreen (clone REA675, Miltenyi Biotec, Bologna, Italy) using a FACS Canto II (BD Biosciences, Eysins, Switzerland) and the data were elaborated using the software FlowJo version 10.8 (FlowJo LLC, Ashland, OR, USA). The titer of plasmatic IgG specific for the SARS-CoV-2 RBD spike was determined using an Access SARS-CoV-2 IgG II assay (Beckman Coulter, Milan, Italy), following the manufacturer’s instructions.

### 2.2. Collection of Salivary Specimens

Saliva specimens were collected at the analysis laboratory of the University Hospital of Ferrara, following the guidelines described in the Human Microbiome Project protocol (Manual of Procedures for Human Microbiome Project, Core Microbiome Sampling, Protocol A, HMP Protocol # 07–001, Version Number: 12.0, 29 July 2010) [27], as previously reported [28]. Subjects were asked not to eat/drink or rinse their mouths for 1 h before sampling, and sample collection was performed under the supervision of a trained operator. Briefly, recruited subjects were requested to stimulate saliva production for 30 s by making chewing motions and gently rubbing the outside of their cheeks. Then, salivary fluids were self-collected into a sterile 50 mL collection tube, opened only just before use. Collected sample volumes ranged from 2 to 5 mL of saliva. 

Immediately after sampling, collected specimens were refrigerated (5 ± 3 °C) and transported within 2 h to the microbiology laboratory of Ferrara University where they were spun by centrifugation for 1 min at 14,000× *g* at 4 °C, split into two aliquots, and then stored at −80 °C until use. 

### 2.3. Analysis of Salivary Anti-SARS-CoV-2 IgA

The presence and amount of salivary anti-SARS-CoV-2 IgAs were evaluated using the commercial quantitative ELISA assay “RayBio COVID-19 S1RBD”, targeting human IgA directed against the receptor-binding domain of the viral spike protein S1-RBD (RayBiotech Life, Peachtree Corners, GA, USA), as previously reported [20]. The assay protocol, originally intended for analysis of serum samples, was first optimized for saliva analysis by testing serial salivary dilutions. Based on the preliminary results, saliva samples were tested at a 1:10 dilution in the sample diluent provided by the assay kit. Each sample and control (including standard curve dilutions, blank, and positive controls) were tested in duplicate, following the manufacturer’s instructions. In parallel, an albumin-coated plate was loaded with the same samples and assayed as a negative control for background subtraction of optical density (OD) values at 450nm with respect to the S1-RBD-coated plate. The mean OD value obtained in the blank wells was further subtracted from all saliva and standard samples. The IgA titer, expressed as Unit/mL, was obtained by interpolation on the calibration curve, and values greater than 21.4 U/mL were considered positive, according to the manufacturer’s instructions.

### 2.4. Statistics 

ANOVA, Kruskal–Wallis and unpaired Student’s *t*-test were used to compare numerical parameters, including age and IgA antibody titer, in the subgroups of vaccinated subjects. The Fisher’s exact test was used to compare the frequency of non-numerical epidemiological parameters (gender distribution, SARS-CoV-2 positivity) between subgroups. The correlation between presence/amount of salivary IgA and age of recruited subjects was calculated using a Spearman correlation analysis. Values of *p* ≤ 0.05 were considered significant.

## 3. Results

### 3.1. Features of Recruited Vaccinated Subjects 

One hundred and five subjects were recruited after receiving an anti-SARS-CoV-2 booster vaccine at the University Hospital of Ferrara (Italy). Demographic features and vaccine characteristics of the enrolled subjects, collected at enrollment, are reported in Table 1. The whole enrolled population (105 subjects) had a mean age of 47.38 ± 13.55 years and included 37 males (35.2%) and 68 women (64.8%). All subjects were recruited between 4 and 9 months after receiving the booster dose. A small fraction of individuals (10/105, 9.5%) had developed a SARS-CoV-2 infection prior to recruitment (COVID(+) subgroup). The COVID(+) subgroup had a mean age of 39.40 ± 9.37 years and included nine women (90%) and one male subject (10%). The remaining COVID(−) population included 95 subjects with a mean age corresponding to 48.22 ± 13.68 years, and 36/95 (37.9%) were males. No statistically significant differences were observed between the COVID(–) and COVID(+) subgroups in terms of BMI, blood CD4^+^ and B cell counts, and time from booster dose, although COVID(+) subjects showed a significantly higher SARS-CoV-2 blood IgG mean titer compared with the vaccine-only subjects (*p* = 0.001), as conceivable. As this study aimed to assess the development of mucosal IgAs following vaccination, the COVID(+) subgroup was excluded from subsequent analyses.

In the vaccinated uninfected subjects (95 total subjects), as summarized in Table 2, 55 individuals (57.9%) received two doses of the recombinant adenovirus-based AstraZeneca (A) vaccine in the primary cycle of vaccination (first and second dose), whereas 40/95 subjects (42.1%) received two doses of the mRNA-based Pfizer-BioNTech (P) vaccine. For the booster vaccination (third dose), 63/95 subjects (66.3%) received the mRNA-based Moderna (M) vaccine; of them, 44 subjects received the booster M vaccine after two doses of the A vaccine (A-A-M subgroup, 46.3%) and 19 after two doses of the P vaccine (P-P-M subgroup, 20%). Instead, 32/95 individuals (33.7%) received a P vaccine as the third dose, with 11/95 subjects receiving it after two doses of the A vaccine (A-A-P subgroup, 11.6%) and 21/95 after two doses of the P vaccine (P-P-P subgroup, 22.1%). Of note, all the subjects belonging to the COVID(+) subgroup had received the A vaccine in the first round of vaccination, followed by the M booster dose in eight of them (A-A-M subgroup, 80%) and by the P booster in two of them (A-A-P subgroup, 20%).

No significant differences in gender distribution were detected by statistical analysis among the vaccine subgroups in the vaccinated population. Some statistically significant age differences were instead observed in the P-P-M group compared with the A-A-M and A-A-P groups (*p* = 0.0004 and *p* = 0.007, respectively), which reflected the specific guidelines introduced for the first vaccination cycle by the Italian Ministry of Health suggesting that using the A vaccine in people less than 40 years old be avoided. In parallel, anti-SARS-CoV-2 IgG titer appeared higher in the P-P-M subgroup compared to the total study group (*p* = 0.01), A-A-M subgroup (*p* = 0.0008), and A-A-P subgroup (*p* = 0.01).

### 3.2. Anti-SARS-CoV-2 Oral IgA analysis

Salivary fluids were self-collected from the recruited subjects and used to assess the percentage of oral IgA positivity and the amount of IgA detected in the vaccinated study population and in the vaccine subgroups, which are reported in Table 3. Overall, 89/95 (93.7%) vaccinated subjects tested positive for anti-SARS-CoV-2 salivary IgA antibodies at the oral level, showing a mean value of 351.5 ± 31.77 U/mL (range 0–1671 U/mL). 

By stratifying the study cohort into four subgroups based on the combination of received vaccines, no statistically significant differences were observed. Specifically, in the A-A-M subgroup, 41/44 (93.2%) subjects showed detectable levels of salivary IgA, with a mean titer corresponding to 320.6 ± 45.80 U/mL (range 0–1671 U/mL). In the A-A-P subgroup, 11/11 (100%) subjects tested positive for salivary IgA presence, with a mean titer corresponding to 416.9 ± 100.6 U/mL (range 95.331–1351 U/mL). In the P-P-M subgroup, 18/19 (94.7%) subjects tested positive for IgA presence, with a mean titer corresponding to 436.3 ± 80.77 U/mL (range 0–1353 U/mL). Last, the P-P-P subgroup showed IgA positivity in 19/21 subjects (90.5%), with a mean IgA titer corresponding to 305.0 ± 58.36 U/mL (range 0–1161 U/mL). No significant differences were observed in the salivary IgA titer among the vaccine subgroups.

**Figure 1:**
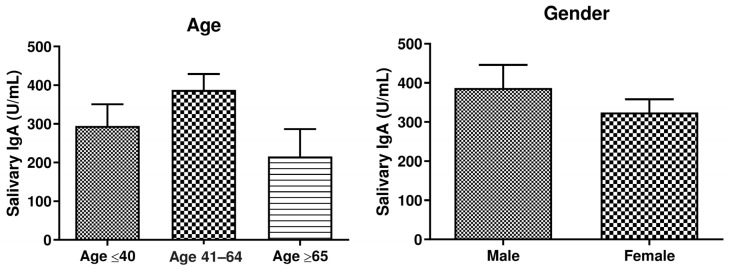
Anti-SARS-CoV-2 oral IgA in enrolled subjects, grouped based on epidemiological features (age, gender). Salivary IgA titer is expressed as mean values (Unit/mL) ± SE. *p*-values calculated using an unpaired Student’s *t*-test.

To assess the eventual influence of the subjects’ demographic features on the vaccine-induced salivary IgA production, we stratified the vaccinated population according to age and gender (Figure 1). The results showed that oral IgA titer was not significantly influenced by age, although, conceivably, elderly subjects (age ≥ 65 years old, y.o.) exhibited lower levels of salivary IgA compared to middle-aged (41–64 y.o.) or young-aged (≤40 y.o.) subjects. Specifically, the mean salivary IgA titer corresponded to 215.6 ± 70.76 U/mL in the elderly group, 387.7 ± 41.04 U/mL U/mL in the middle-aged group, and to 294.9 ± 55.57 in the group including people aged ≤ 40 years. 

Similarly, no significant differences were observed relative to gender, despite a slight tendency toward a lower response that was observed in the male group compared to the female group (337.3 ± 53.41 U/mL vs. 360.1 ± 39.75 U/mL, respectively). 

Of note, by contrast, the small subgroup of subjects who developed a SARS-CoV-2 infection after vaccination exhibited mucosal IgA in all of their saliva specimens (10/10, 100%), with a mean titer significantly exceeding that detected in the vaccine-only subjects (796.2 ± 298.6 U/mL), as expected (*p* = 0.001).

**Figure 2:**
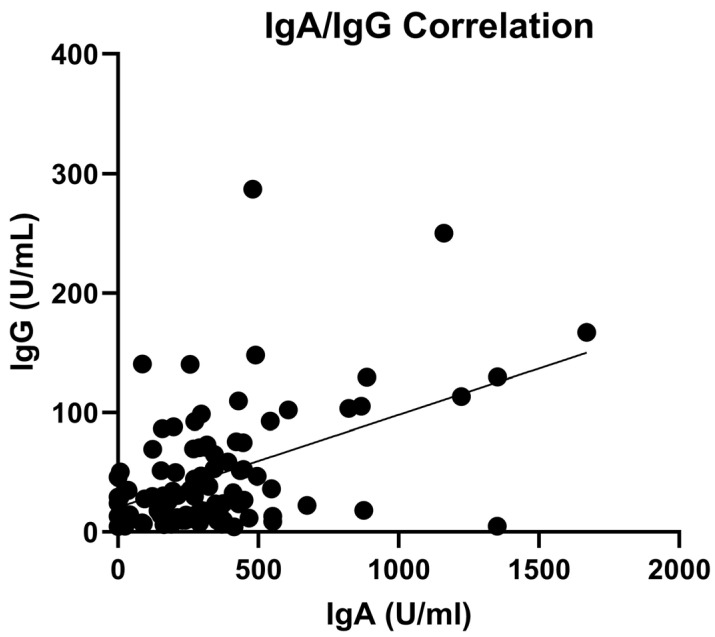
Correlation between anti-SARS-CoV-2 salivary IgA and serum IgG. Antibody titer is expressed as AU/mL. Black dots represent the analyzed samples. Correlation coefficient was calculated using a Spearman’s test.

Next, we analyzed the eventual correlation between mucosal IgA development and other individual parameters, including BMI, blood CD4^+^ and B cell counts, anti-SARS-CoV-2 blood IgG titer, and time from booster dose. No correlation was evidenced between salivary IgA titer and any of the analyzed parameters, except for a strongly positive correlation between the concentration of mucosal oral IgA and that of serum anti-SARS-CoV-2 IgG antibodies (*p* < 0.0001), depicted in Figure 2.

**Figure 3:**
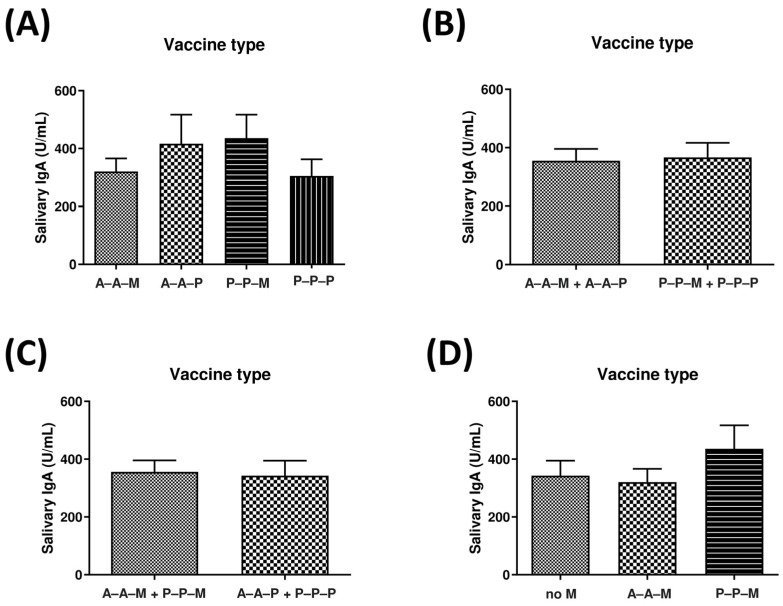
Anti-SARS-CoV-2 oral IgA in enrolled subjects, grouped based on the combination of administered vaccines (AstraZeneca, A; Pfizer-BioNTech, P; Moderna, M). (**A**) Subgroups based on the four vaccines’ combination included in the study; (**B**) subgroups who received A or P as the first vaccine; (**C**) subgroups who received M or P as the booster vaccine dose; (**D**) subgroups who received or not M as the booster dose. Salivary IgA values are expressed as mean values (unit/mL) ± SE. The indicated *p*-values were calculated using an unpaired Student’s *t*-test with Welch’s correction.

By subdividing the study population according to the type of vaccine combination received, only a few non-significant differences emerged (Figure 3A). In detail, the salivary IgA titer appeared slightly higher in subjects that received a booster M dose after a P-P primary cycle (436.3 ± 80.77 U/mL), followed by those that received the A-A-P combination (416.9 ± 100.6 U/mL), whereas the A-A-M and P-P-P subgroups exhibited a lower IgA titer (320.6 ± 45.80 U/mL and 305.0 ± 58.36 U/mL, respectively). However, the comparison between the P-P-M and P-P-P subgroups did not indicate any statistically significant difference (*p* = 0.09). 

Again, no significant differences were detectable between subjects that received two doses of the A vaccine in the first round of vaccination (A-A-M plus A-A-P subgroups) compared to those that received two doses of the P vaccine (P-P-M plus P-P-P subgroups) (339.9 ± 41.69 vs. 367.4 ± 49.57, respectively, *p* = 0.34) (Figure 3B). Similarly, no statistically significant difference was observed between subjects who received the M vaccine as the booster dose (A-A-M plus P-P-M subgroup) compared to the others (A-A-P plus P-P-P subgroup) (*p* = 0.43) (Figure 3C). Also, the comparison between the subjects who did not receive an M vaccine dose (no M) with the vaccinees that received an M booster dose (A-A-M and P-P-M groups) (Figure 3D) showed a higher IgA level in the P-P-M group compared to both the A-A-M and no-M groups, although the differences were not statistically significant (*p* = 0.11 and 0.17, respectively).

**Figure 4:**
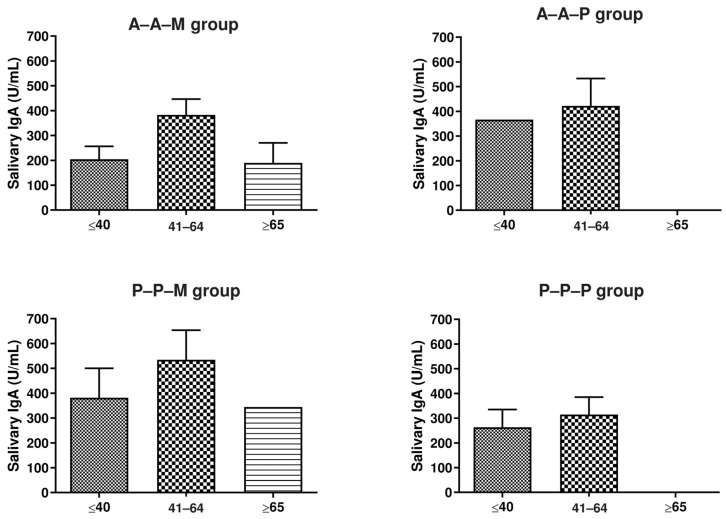
Anti-SARS-CoV-2 salivary IgA in enrolled subjects, stratified based on the combination of received vaccines (AstraZeneca, A; Pfizer-BioNTech, P; Moderna, M) and subdivided based on age into young (≤40 y.o.), middle-aged (41–64 y.o.), and elderly (≥65 y.o.) subgroups. Salivary sIgA values are expressed as mean values (U/mL) ± S.D. The indicated *p*-values were calculated using an unpaired Student’s *t*-test with Welch’s correction.

Last, since age is considered to be an important factor in the development of an effective and sustained immune response, we performed the same analyses after stratifying the vaccine subgroups according to the three age ranges considered in the analysis of the total study population, i.e., young subjects (≤40 y.o.), middle-aged subjects (41–64 y.o.), and senior subjects (≥65 y.o.) (Figure 4). Based on these analyses, no statistically significant differences emerged between age subpopulations. Of note, the number of subjects included in the elderly group was very low, six out of ninety-five, of which five were in the A-A-M subgroup (mean age 69.2 y.o.) and one was in the P-P-M subgroup (66 y.o.). 

## 4. Discussion

Several published pieces of clinical data support the effectiveness of booster vaccination in preventing a worse disease outcome and COVID-19-related deaths [12], and increasing attention is being given to the impact of vaccination on the development of the immune response at mucosal sites, representing the primary route of viral entry and infection [13,14]. 

Toward this direction, we previously showed an inverse correlation between salivary IgA levels and COVID-19 severity [19] and further reported the presence of mucosal IgA in the conjunctival fluid of infected patients [16], showing that ocular IgA titers correlated with a milder course of the disease [16].

Based on these observations, we became interested in assessing the development of mucosal responses following anti-SARS-CoV-2 vaccination. We recently demonstrated that the primary cycle of SARS-CoV-2 vaccination, consisting of the administration of two doses, was able to elicit a specific IgA response at the eye level [20], and subsequent reports also confirmed these data at the oral level [25,26]. More specifically, the administration of two doses of the BNT162b2 mRNA Pfizer vaccine was shown to induce early but weak salivary IgA immunity (two weeks after the second dose), which increased and persisted for longer times (until eight months after the second dose) [24]. Of note, studies also showed that booster vaccination with BNT162b2 could elicit higher titers of both IgG and IgA in serum and saliva compared with the primary vaccination cycle, although it was not effective at inducing mucosal immunity against the emerging Omicron BA.1 VOC [24]. Other studies highlighted that the oral IgA levels elicited by booster vaccination were significantly lower compared to after SARS-CoV-2 infection, suggesting that the vaccines used were not as effective at inducing protective mucosal responses [8].

Since the reports available so far on the mucosal IgA response following booster vaccination refer almost exclusively to the BNT162b2 (Pfizer-BioNTech) vaccine, in this study, we wanted to characterize the development of oral IgA mucosal immunity in response to the different combinations of COVID-19 vaccine formulations used for the vaccination campaign in Italy, including the A (Astra-Zeneca), M (Moderna), and P (Pfizer-BioNTech) vaccines. To this purpose, 105 subjects vaccinated with an anti-SARS-CoV-2 booster dose were enrolled in the study and analyzed for the presence and amount of salivary IgA. Of them, ten subjects developed a SARS-CoV-2 infection despite vaccination and were thus considered separately. 

In the cohort including uninfected vaccinated subjects (consisting of 95 subjects), specific anti-SARS-CoV-2 salivary IgA was detectable in 93.7% of salivary samples, showing that all vaccine combinations were able to stimulate the development of a mucosal response at the oral level, corresponding to an overall mean concentration of salivary IgA of 351.5 ± 31.77 U/mL. By subgrouping the study participants according to demographic features and vaccine types, no statistically significant differences were observed. Specifically, a slightly lower response was detected in the female group compared to the male group and in the elderly group compared to the other age groups, likely due to the constitutive senescence of the immune system during advanced age. However, the low number of elderly subjects enrolled in this study and the relatively low age (<70 y.o.) renders it difficult to draw conclusions about the eventual impact of aging on the mucosal IgA response at the oral level. Rather, the overall data highlight the long-term presence of mucosal antibodies in all the vaccine regimens and with little variation relative to age. 

Interestingly, a higher salivary IgA response correlated with a higher induction of blood anti-SARS-CoV-2 IgG, evidencing that individuals able to develop a good mucosal response also develop an effective IgG response and suggesting that the measurement of IgA in saliva could be predictive of an efficient good systemic response.

Of note, the salivary IgA titer was more than doubled in subjects who had developed a SARS-CoV-2 infection despite vaccination compared to those who only entered in contact with the spike viral protein through vaccination (796.2 ± 298.6 U/mL vs. 351.5 ± 31.77 U/mL, *p* = 0.001), confirming that a naturally occurring infection is more effective than current available vaccines at inducing oral IgA production, as previously reported [8]. This also suggests that vaccines could be potentiated toward a better ability to elicit mucosal immunity to provide a more effective early control of virus replication. 

To identify any difference in the ability to elicit a mucosal IgA response, we dissected the study cohort based on the type of vaccine combination administered. However, no statistically significant differences were observed between the different vaccine formulations. Specifically, no differences were detected between vaccinee subgroups in terms of the frequency of positivity and titer of oral IgA, although subjects who received the mRNA-1273 (Moderna) vaccine as the booster dose after two P vaccines showed a slightly higher IgA titer compared to the others. However, the difference did not reach statistical significance (*p* = 0.11), likely due to the relatively low number of subjects. Despite this, these data are in line with what was previously observed in conjunctival fluid of vaccinated and infected patients and suggest that studying the formulation and vaccination schedule of anti-COVID-19 vaccines may be important in the development of protective mucosal responses. Toward this goal, it has been recognized that better mucosal immunity would be needed to control COVID-19 infection in the future [29], and our results encourage further studies in this direction, such as those evaluating the ability of the new bivalent vaccines at inducing efficient/neutralizing mucosal IgA antibodies, as no data are currently available. Since it was reported that the two bivalent formulations of mRNA vaccines original/omicron BA.1 and original/BA.4–5 (Spikevax and Comirnaty, respectively) were able to elicit a higher IgG response against both the Omicron BA.1 and BA.4–BA.5 variants [30,31,32], it would be of value to evaluate their potential as mucosal IgA inducers. Also, it would be of interest to explore the prognosis potential of anti-SARS-CoV-2 IgA in saliva, based on the results showing that the oral IgA amount paralleled the IgG titer in blood. 

Consistent with our data, a recent study reported the ability of different COVID-19 vaccine types to elicit similar salivary anti-SARS-CoV-2 IgG [33]. In addition, other studies showed that IgA response in saliva mostly falls to baseline after 6 months post-infection and that vaccination can reactivate the mucosal response, suggesting that vaccine platforms inducing better mucosal immunity would be needed to control COVID-19 infections in the future [29].

Limitations of this study include the relatively low number of enrolled subjects and the lack of multiple sampling to assess the trend of oral IgA over time following vaccination. As well, only the concentration of salivary IgA was investigated, while it would be interesting to assess the neutralizing ability of the detected antibodies in order to correlate the protective activity with effective mucosal IgA action. Finally, some comparisons were not possible due to the fact that A vaccine usage was discontinued in young people based on the recommendations of the Italian Ministry of Health.

However, the collected data indicate that the available anti-COVID-19 vaccines can drive the development of mucosal responses in vaccinated subjects, although the IgA titer is significantly lower compared to the level in naturally infected individuals. Consistent with the results reported in other studies [34], our data suggest that actions may be undertaken to improve the mucosal response in future vaccine formulations and to include new vaccination strategies focused on mucosal administration.

## 5. Conclusions

Overall, almost the totality of the vaccinated population develops an oral IgA response upon intramuscular vaccination with different types of COVID-19 vaccines (93.7%). A tendency to develop a higher oral IgA response was observed in subjects receiving the Moderna vaccine as the booster dose following two doses of the Pfizer vaccine in the primary vaccination cycle, confirming previous results obtained in the conjunctival fluid. This first study paves the way for other future comparative studies aimed at assessing the ability of different vaccine formulations to induce mucosal immunity and to correlate such induction with vaccine effectiveness in providing prevention of infection and protection from the disease. Our data also suggest that the oral cavity may represent an accessible site to assess vaccine-elicited immune responses as the development trend of salivary IgA reflects that of the systemic humoral response. In addition, salivary IgA monitoring may represent a useful tool for driving forward vaccine design, potentially leading to novel strategies for vaccine administration and boosting.

## Figures and Tables

**Table 1:** Demographic and clinical features of the study population.

Parameters	Enrolled Subjects		
	Total	COVID(−)	COVID(+)
Participant No.	105	95	10
Age (years)	47.38 ± 13.55	48.22 ± 13.68	39.40 ± 9.37
Gender (male)	37 (35.2%)	36/95 (37.9%)	1/10 (10%)
BMI	24.04 ± 3.60	24.13 ± 3.70	23.19 ± 2.39
CD4^+^ cell No.	718.9 ± 244.0	714.6 ± 251.6	757.7 ± 165.3
B cell No.	175.9 ± 90.71	175.0 ± 93.39	184.1 ± 64.57
Anti-SARS-CoV-2 IgG	52.97 ± 55.64	47.78 ± 50.84	102.3 ± 76.07 *
Time from booster	5.33 ± 0.80	5.32 ± 0.82	5.50 ± 0.71

Age results are reported as mean age (years) ± S.D. BMI, body mass index. CD4^+^ and B cell No. are reported as cell number per µL of blood. Anti-SARS-CoV-2 IgG titer is reported as mean serum concentration ± S.D. (AU/mL). Time from booster is indicated as number of months. * Statistically significant difference.

**Table 2:** Features of the study group subdivided relative to the type of received vaccine.

Parameters		Vaccine Subgroups			
	Total (*n* = 95)	A-A-M (*n* = 44)	A-A-P (*n* = 11)	P-P-M (*n* = 19)	P-P-P (*n* = 21)
Age (years)	48.22 ± 13.68	52.05 ± 12.51	53.09 ± 9.03	38.53 ± 14.84 *	46.43 ± 12.91
Gender (male)	36/95 (37.9%)	19/44 (43.2%)	4/11 (36.4%)	4/19 (21.1%)	9/21 (42.9%)
BMI	24.13 ± 3.70	24.16 ± 4.06	25.18 ± 1.85	23.06 ± 3.40	24.51 ± 3.86
CD4^+^ cell No.	319.2 ± 180.9	317.8 ± 144.1	287.5 ± 128.2	302.4 ± 129.7	269.8 ± 126.5
B cell No.	175.0 ± 93.39	175.5 ± 107.7	148.3 ± 60.67	172.9 ± 96.78	194.3 ± 62.11
Anti-SARS-CoV-2 IgG	47.78 ± 50.84	34.46 ± 39.05	25.17 ± 22.48	81.49 ± 65.84 *	57.03 ± 53.61
Time from booster	5.32 ± 0.82	5.32 ± 0.83	5.27 ± 0.47	5.42 ± 1.07	5.24 ± 0.70

Age results are reported as mean age (years) ± S.D. Vaccine types: A, AstraZeneca; P, Pfizer-BioNTech; M, Moderna. BMI, body mass index. CD4^+^ and B cell No. are reported as cell number per µL of blood. Anti-SARS-CoV-2 IgG titer is expressed as mean serum concentration ± S.D. (AU/mL). Time from booster is indicated as number of months. * Statistically significant difference.

**Table 3:** Anti-SARS-CoV-2 IgA positivity and titer in saliva specimens.

Oral IgA	Subgroups				
	Total (*n* = 95)	A-A-M (*n* = 44)	A-A-P (*n* = 11)	P-P-M (*n* = 19)	P-P-P (*n* = 21)
Positivity %	93.7	93.2	100	94.7	90.5
IgA titer (U/mL)	351.5 ± 31.77	320.6 ± 45.80	416.9 ± 100.6	436.3 ± 80.77	305.0 ± 58.36

IgA titer is expressed as mean saliva concentration ± S.D. (U/mL); A, AstraZeneca vaccine; P, Pfizer-BioNTech vaccine; M, Moderna vaccine.

## Data Availability

All data generated during this study are included in the manuscript.
